# Combining online and offline peer support groups in community mental health care settings: a qualitative study of service users’ experiences

**DOI:** 10.1186/s13033-020-00370-x

**Published:** 2020-05-29

**Authors:** Monica Strand, Lillian Sofie Eng, Deede Gammon

**Affiliations:** 1 Department of Digital Health Research, Division of Medicine, Oslo University Hospital, Oslo, Norway; 2 Department of Mental Health Research and Development, Division of Mental Health and Addiction, Vestre Viken Hospital Trust, Drammen, Norway; 3 Norwegian Center for eHealth Research, University Hospital of North-Norway, Tromsö, Norway

**Keywords:** eHealth, Mental health, Recovery, e-Recovery, Peer support, Social support, Qualitative research, Participatory research

## Abstract

**Background:**

Peer support for people with long-term mental health problems is central to recovery-oriented approaches in mental health care. Peer support has traditionally been conducted offline in face-to-face groups, while online groups on the Internet have increased rapidly. Offline and online peer support groups are shown to have differing strengths and weaknesses. However, little is known about how combining the two formats might be experienced by service users, which this paper aims to illuminate.

**Methods:**

In this exploratory and descriptive study, a recovery-oriented Internet-based portal called ReConnect was used by service users in two mental health communities in Norway for 6–12 months. The portal included an online peer support group which also facilitated participation in local offline peer support groups. Both group formats were moderated by an employed service user consultant. Qualitative data about service users’ experiences were collected through focus groups and individual interviews and inductively analyzed thematically.

**Results:**

A total of 14 female service users 22–67 years of age with various diagnoses participated in three focus groups and 10 individual interviews. Two main themes were identified: (1) balancing anonymity and openness, and (2) enabling connectedness. These themes are further illustrated with the subthemes: (i) dilemmas of anonymity and confidentiality, (ii) towards self-disclosure and openness, (iii) new friendships, and (iv) networks in the local community. Three of the subthemes mainly describe benefits, while challenges were more implicit and cut across the subthemes. Identified challenges were related to transitions from anonymity online to revealing one’s identity offline, confidentiality, and barriers related to participation in offline peer support groups.

**Conclusions:**

This study suggests that online and offline peer support groups complement each other, and that combining them is mainly described as beneficial by service users. Identified benefits appeared to arise from service users’ options of one format or the other, or that they could combine formats in ways that suited their individual values and comfort zones. Moderation by a trained service user consultant appeared essential for both formats and can be used systematically to address identified challenges. Combining online and offline peer support groups is a promising concept for facilitating recovery-oriented care and warrants continued research.

## Background

Peer support for people with long-term mental health problems has shown promise in facilitating personal recovery processes as well recovery-focused changes in services [1–7]. Peer support in mental health care, whether provided one-on-one or in groups, is defined as “a system of giving and receiving help founded on key principles of respect, shared responsibility, and mutual agreement on what is helpful” [6] and involves people with lived experiences of mental health problems supporting others in their recovery process [4, 8]. An underlying premise for peer support, is that individuals facing a similar life event or health-related problems are in a unique position to understand one another in ways that one’s professionals, friends and family may not [9]. For recovery-oriented approaches to mental health, where peer support is an integral component, recovery has been defined as a personal process comprising of five dimensions: connectedness to others and the community; hope and optimism about the future; identity building beyond being a patient and towards a positive sense of identity without stigma; meaning in life; and empowerment [10]. Peer support is identified as a key resource in promoting hope and the belief that recovery is possible for persons with similar mental health problems [8]. It is also integral to the increased emphasis on the relational aspects of recovery [11–13]. Peer support groups are often moderated by peers or professionals, or a combination of both and can take different forms, for example, run independently in the community or as an adjunct to ongoing mental health care [14–16]. In peer support groups the social exchanges of mutual support and experiential knowledge are believed to be central to therapeutic processes [1, 17], including the therapeutic effect of helping others [14, 18]. However, the complexity of researching the diverse types and contexts of peer support, whether provided individually or in groups, has hampered efforts to amass conclusive evidence of its effectiveness [16, 19]. Research nevertheless suggests that peer support can improve outcomes such as quality of life and hope [4, 20], increase social network and wellness [21], and reduce treatment costs and rates of re-hospitalization [17].

While much of the literature on peer support is based on offline face-to-face settings, online peer support has evolved rapidly in recent years [22, 23], also for mental health issues [24] exemplified through different types of interventions and target groups [25–27]. An online support group or community is defined as “any virtual social space where people come together to get and give information or support, to learn, or to find company” [28]. Predictors of participation in online peer support are: (i) limited access to adequate support within traditional social network(s), (ii) living with health-related stigma, (iii) perceived similarity and credibility of support providers, (iv) and convenience and other features of computer-mediated communication [23]. Communication in online peer support groups is often conducted through e-mail, bulletin boards, or specific software for live interaction with other group members (ibid.) with 24/7 accessibility regardless of location. Online peer support could be either public and open or closed and private [14]. Several studies have found that people are more willing and feel more comfortable sharing sensitive information or asking sensitive questions on the Internet [29, 30].

Studies of online peer support groups find many positive aspects that resemble those of offline peer support groups, including; social connectedness [31–33], help cope with day-to-day challenges and stigma reduction [33], and facilitate insights into health care decisions [32], empowerment [34], and recovery processes [33, 35]. Additionally, online peer support groups have been found to provide emotional support, insights and experiences about living with mental health problems which are not typically available through traditional mental health care [36]. Online peer support groups that are moderated either by peers or professionals have been found to have higher levels of retention, engagement, acceptability, perceived social support and efficacy than for online peer support without moderators [37]. The anonymity offered online is suggested to be important for an open and non-judgmental atmosphere [38, 39]. Disinhibition and self-disclosure, which is often associated with online communication, is also evident in online peer support groups [40] which might be related to that some find it easier to express ones’ ‘true-self’ online compared to offline [41]. This type of atmosphere has also been found to lower thresholds for participation for those concerned with stigma, or who experience from social anxiety [36].

Challenges related to online peer support groups have also been reported. While the social relationships that have evolved through online communities can migrate to the real world [38], it is also suggested that online interactions might undermine exposure to real-life social exchanges and relationships which can be decisive for recovery processes and stigma reduction [36]. Concerns about online peer support include risks for excessive use leading to a decrease in offline interactions [42, 43] and reported negative effects such as social avoidance and excessive dependency of online peer support [44, 45]. It remains unclear whether feeling less alone, learning from peers, and gaining confidence from interacting with others online translate into tangible and meaningful improvements in recovery, employment, or mental and physical wellbeing in the real world [46].

Implementation of recovery-oriented practices in mental health is demonstrated to be challenging [47–50]. While offline peer support is established as integral to recovery-oriented practice, research into the role that online peer support might play in shaping recovery processes in mental health care is still in its formative stages [51, 52]. Online and offline formats for peer support groups have qualities that are potentially complementary and research into the possible outcomes of interactions between the two formats has been called for [23]. In the current study we explored use of both formats when introducing ReConnect to two mental health communities in Norway. ReConnect is a recovery-oriented Internet-based intervention designed to support both personal recovery processes and collaboration with health providers for service users in mental health care, referred to as an e-recovery portal [51, 53]. The portal provided service users online peer support, and was also used to organize monthly offline peer support in the local community. We sought insights into service users’ experiences of combined peer support relative to their recovery processes by posing the following research question: With a particular focus on potential benefits and challenges, how do service users describe their experiences of combining online and offline peer support groups?

## Methods

### The e-recovery portal—ReConnect

ReConnect consists of a secure messaging system between service users and health providers, an online peer support group (forum), and a toolbox with a set of resources that support service users in articulating and working with various aspects of their lives, such as setting goals and planning activities. Other resources include: a network map; a crisis plan; different exercises related to mindfulness, coping, and symptom management; a medication overview; information about user involvement, working relationships, personal recovery, and how to use ReConnect; and links to local activities and service users’ organizations. ReConnect was self-managed by service users in that they had exclusive access to all content, while their health providers could remotely access parts of the content generated by service users. ReConnect was designed to support collaboration between service users and their health providers during or between consultations. The forum was asynchronous and all participants could initiate topic threads. Service users from two communities participated in the same forum. In addition, local offline peer support groups (ReConnect-cafés) where service users could meet face-to-face were held separately once a month in the participating communities. Both the ReConnect-cafés and the forum were moderated by an employed service user consultant with lived experience of mental health problems and with training in peer support.

Over a 6-month period, 29 service users participating in a mixed methods study about use of ReConnect wrote 524 forum posts and viewed them 1870 times [51]. Seventeen service users participated in a total of 12 ReConnect-cafés (range 3–9 participants per meeting), six per site, over a period of 8 months. All of the participants viewed forum posts, while 19 wrote at least one post.

### Study methodology and design

This explorative and descriptive study with elements of participatory approaches [54–57] studied services users’ experiences of use of ReConnect as an adjunct to ongoing mental health care. Participants used the portal for at least 6 months and at most 12 months. The service user consultant was in addition to moderator of the peer support groups also part of the research team as a co-researcher. She contributed to refinement of research questions and methods, coordinating input from a network of service users about our emerging findings, and was actively involved in dissemination of findings. She also introduced topics relevant for recovery processes in both formats of peer support. Study participants were invited to give feedback about the research process, e.g. about the interview guide, implementation of the portal and the study’s findings as they unfolded (further elaborated below). Conscientious of power imbalances in collaborations between service users and researchers [57], a number of steps were taken to foster confidence in the important role of the service users and for building rapport within the research team [58]. Data were generated in focus groups [59, 60] and individual interviews [61]. The focus groups were held approximately 3 months into the study so that discussions among participants could also serve to stimulate use and their own recovery processes in the remainder of the study. The individual interviews were used to generate more personal and detailed information [62], and were held after 6 to 8 months of study participation, when participants had more experience of using ReConnect (further detailed below).

### Setting

Norway has universal health care that is publically funded as part of the national budget through general and earmarked grants. The municipalities are responsible for providing primary health care and social services, while the Regional Health Authorities provide specialist services (e.g. acute wards, district psychiatric centers). As used in this paper, the word “participating communities” refers to care at primary and specialist care levels provided to residents of two municipalities in Norway: one small community in the north with about 5700 inhabitants within an area of 1493 km^2^, and one large community on the outskirts of the capital with about 59,000 inhabitants within an area of 100 km^2^. These two communities were chosen to ensure breadth in size and location of communities in a Norwegian setting. Both communities expressed commitments to policies promoting eHealth, user involvement, and collaborative practices [51].

### Recruitment and participant inclusion

Participants in the current study were recruited among 29 service users participating in a mixed methods study about the use of ReConnect among service users in mental health care including collaboration with their health providers [51]. Inclusion criteria for service users in the mixed methods study were: over 18 years of age, had received mental health services for at least 6 months prior to inclusion, and had expectations of needing services at least 6 months forward, Internet access with a public key solution for secure electronic identification, and at least one of their health providers willing to participate in the study. For the current study, service users in the mixed methods study were invited by the research team and/or health providers to take part in focus groups and/or individual interviews about their experiences with the use of ReConnect. For the focus groups, all of the included service users were invited to participate. For the individual interviews, we intentionally sought participants who had experience of using ReConnect, defined as having logged on > 15 times. In both the focus groups and individual interview we sought range of participants in terms of age, gender, mental health problems, and types of ongoing mental health care support.

### Focus groups and individual interviews

The interview guide for the focus groups consisted of questions about ReConnect relative to working relationships and recovery processes (see Additional file 1), the latter of which is of relevance for the current study. In line with the explorative nature of the study, the questions were few and open-ended in order to stimulate group dialogue [59, 60] about the role that ReConnect, including online and offline peer support, might play in their recovery processes. Participants were given the opportunity to elaborate on subjects they considered relevant and important. Prompts that could encourage openness, and elicit examples and detail were used frequently. The focus groups were conducted by the first author who is a trained registered nurse with clinical experience from the field, and the second author who was the study’s service user consultant, and who had first-hand experience of mental health problems and recovery at both primary and specialist levels of mental health care. The focus groups lasted for approximately 90 min.

The individual interviews sought to elicit more in-depth personal experiences relative to the same topics as in the focus groups, also based on semi-structured interview guides with open-ended questions (see Additional file 2). Individual interviews were conducted by the first author, with the exception of one individual interview conducted by the second author, and lasted approximately 60 min. All focus groups and individual interviews were conducted in Norwegian.

### Thematic analysis

The focus groups and individual interviews were audio-recorded and transcribed verbatim. Data analysis was aided by NVivo software version 11. The data were analyzed by applying a six-phase thematic analysis for identifying, analyzing and reporting patterns within the data [63]. The main goal during the analysis was to inductively sort the material into overarching themes and subthemes across the entire data set, guided by the research question [63]. The first author led the analysis process, involving the other authors in identifying, discussing and resolving potential differences in coding and interpretive practices (e.g. detail, level of abstraction). This facilitated multiple perspectives in the process of interpreting the data. In the first phase, the first author familiarized herself with the data, noted initial ideas, and assigned and discussed preliminary descriptive codes. In the second phase, relevant extracts of the data (i.e. part or all of a sentence, or a small paragraph about one particular subject identified in the data related to the research question) were systematically identified and entered into NVivo nodes (codes) across the entire data set. The third phase consisted of collating related codes into preliminary themes and gathering all data relevant to each potential theme. In the fourth phase, the themes were reviewed and adjusted relative to overlaps or inconsistencies both to the coded extracts and the entire data set. With the goal of generating clear definitions and names for each theme, the fifth phase refined the wording of each theme and the overall story of the analysis. Finally, in the sixth phase the authors produced the report by selecting vivid and compelling quotes to use in a final analysis relating back to the research question. These phases are described sequentially, but in practice, they were conducted as a recursive process [63], moving back and forth as needed. Thus, consistent with inductive qualitative analysis, the themes and sub-themes evolved continuously during the analysis [62].

After finalizing the report, the third author, a native northern American who is fluent in Norwegian, translated the selected quotes to English. To assess the validity of translations, as well as to backtrack to the dataset when context was needed to ensure that the translation captured the quotes’ meaning, the original quotes were kept alongside the translations [64].

Applying elements of participatory approaches [54–56], participants were invited to give feedback on tentative written and oral summaries of the data through secure messaging, in ReConnect-cafés, or in the individual interviews. The project also conducted a workshop with the aim of eliciting service users’ reflections about the preliminary findings of the focus groups interviews. This not only facilitated the participants’ contribution to understanding and validating the data, but it also facilitated sharing ideas about how to use ReConnect relative to their recovery process in the remaining participation period. Because of this process, experiences on how ReConnect facilitated friendship among the participants was underlined, inspiring the researchers to highlight this as an independent theme.

### Ethics

The study was approved by the Regional Committees for Medical and Health Research Ethics in Norway and the Privacy Protection Committees at the participating sites. Participants signed an online consent form with information about the study which was repeated verbally at the time of the focus groups and individual interviews. Participants consented to use ReConnect exclusively for non-emergency purposes, and to use ordinary channels for acute needs. Participants were given information about security procedures and recommendations for ensuring privacy including how to safeguard anonymity and confidentiality in the forum. Additionally, the participants were registered under a self-selected pseudonym to preserve anonymity. Also, the participants were asked to safeguard confidentiality about information gained at focus groups and in the forum as well as in the ReConnect-cafés. Participants were regularly reminded of the actions they needed to take if they wanted to remain anonymous (i.e. refrain from sharing detailed personal information about oneself in one setting which could lead other participants to discovering one’s identity in another setting).

Additional efforts were made to foster trust and safety among participants, largely by following ethical guidelines for recovery-oriented approaches in mental health care [65]. For example, whether the participants met either online or offline, the research team focused on experiences related to personal recovery topics such as connectedness and empowerment [10] as they related to their uses of ReConnect. When participants themselves raised sensitive issues, whether they were relevant to the research or not, they were heard and acknowledged while at the same time efforts were made to find positive perspectives on what was shared. Regardless of the content expressed by a participant in the forum or in the ReConnect-cafés, the service user consultant found something honest and positive to acknowledge (e.g. I think you are brave to be dealing with this; I admire how you can write so simply about something so difficult; I love your sense of humor). Never once was there a need to censor the forum.

## Results

### The participants

A total of 14 female service users from both primary and specialist levels of mental health care participated in 3 focus groups and 10 individual interviews. Eleven service users participated in the focus groups (range 2–6 participants), while 10 servicer users participated in the individual interviews. Seven of the service users participated in both focus groups and individual interviews, while seven service users participated either in the focus groups or in the individual interviews.

The service users were females from 22 to 67 years of age, and reported various mental health diagnoses (see Table 1).

**Table 1:** Characteristics of participants in the focus groups and individual interviews

	Focus groups (N = 11)		Individual interviews (N = 10)	
	n	(%)	n	(%)
Characteristics				
Age (years), median (range)	45	(22–63)	47	(24–67)
Gender				
Female	11	(100)	10	(100)
Male	0	(0)	0	(0)
Site				
Large community (59,000 inhabitants)	6	(55)	6	(60)
Small community (5700 inhabitants)	5	(45)	4	(40)
Primary care level	5	(45)	6	(60)
Specialist care level	4	(37)	3	(30)
Both levels	2	(18)	1	(10)
Diagnosis				
Depression	6	(55)	7	(70)
Bipolar disorder	2	(18)		
Generalized anxiety	2	(18)	1	(10)
Posttraumatic stress disorder	2	(18)	3	(30)
Schizophrenia	1	(9)	1	(10)
Schizoaffective disorder	1	(9)	1	(10)
Phobic anxiety	1	(9)	1	(10)
Panic anxiety	1	(9)	3	(30)
Drug addiction			1	(10)
Mania			1	(10)
Others	2	(18)	3 (30)	
Number of diagnoses, median (range)	1	(1–5)		1 (1–7)

The thematic analysis generated two main themes that described experiences of combined peer support groups: (1) balancing anonymity and openness; (2) enabling connectedness. The themes and their four subthemes are presented below.

### Balancing anonymity and openness

The combined access to peer support groups online and offline gave rise to the first main theme which highlights participants’ descriptions of transitioning between different levels of anonymity and degrees of self-disclosure.

#### Dilemmas of anonymity and confidentiality

Both the positive and negative aspects of online anonymity in the forum were described by participants as having implications for their face-to-face interactions in the ReConnect-cafés. They reported gradual transitions between different degrees of anonymity in the two formats: from total anonymity online, to revealing online one’s local community, to meeting face-to-face, to making one’s online identity known to peers face-to-face.

For some, anonymity in the online forum made it easier to share sensitive and personal issues in difficult times. In response to the interviewer’s question about the high activity observed in the forum, one responded thus:

*It has to do with the anonymity* […]*. It’s often easier to get things across when you’re sitting and writing compared to when you’re sitting and talking, and you know it’s safe. No one other than us [forum participants] that can see it. It’s really nice* […]*. And you know that if you meet one of the girls at the mall, then they don’t know it’s you.* [Individual interview]

For others, anonymous communication was also described as difficult and impersonal, representing a barrier to openness and sharing in the online forum. One participant described:

*I feel like I’m sitting and talking to … I don’t know who. You sort of talk out into the air. Then the response comes from the air. You don’t know who they are. If feels so unbelievably impersonal.* [Focus group]

The participants’ widely varying experiences of anonymity appeared to be reflected in shared dilemmas about how to maintain the benefits, while minimizing the challenges of combining the formats. Concerns with maintaining confidentiality, a concern also emphasized by the research team, seemed to complicate paths towards resolving the dilemmas, especially when participants had become acquainted through the ReConnect-cafés. Handling confidentiality gave cause concern for some, especially when participants had become acquainted through the ReConnect-cafés. Concern about protecting each other’s identity led some to avoid revealing things in the forum that they had learned from face-to-face-meetings so as not to compromise anonymity online. Not knowing each other’s identity made online communication feel unimportant or superfluous. On the other hand, knowing each other’s identity could also represent a barrier for sharing delicate issues online e.g. about difficult group dynamics experienced at ReConnect-cafés. Also, sharing seemingly innocent information online (e.g. today’s weather) gave cause for concern that identifying information could be pieced together when meeting face-to-face. Moreover, not knowing each other’s forum identities made it reportedly awkward to initiate conversations about topics discussed online when meeting face-to-face. As a way of resolving these challenges, being able to share their identities without compromising others’ identities were advocated. Another suggestion was to offer closed threads for those who had revealed their identities. Underlining these discussions was an expressed acknowledgement of differing needs and preferences, and the difficulties in finding a single solution that would be ideal for everyone. Having different options that allowed each to explore one’s own needs and preferences, and adapt one’s use over time, emerged as something participants valued.

#### Towards self-disclosure and openness

Participants described differing degrees of self-disclosure and that openness about their own situation evolved during the study period, partly due to inspiration from peers, as well as discovering their own preferences. The service user consultant’s role of providing honest and positive responses (see “Ethics”) was reflected in participants’ descriptions of the groups as safe and supportive, and thus also an arena where one could be open.

Openness was initiated by online interactions that were followed up in face-to-face café encounters. Feelings of shame and abnormality were reportedly eased when reading about others with similar experiences, thus facilitating greater self-disclosure and openness online but also offline. One of the participants elaborated:

*Those who have come in* [to the online forum] *see that they get support through what others write, and maybe they feel like … “Hey, I’m not the only one with problems.” And dare to write more about themselves. Not so afraid of being identified. They see that it’s not something to be ashamed of. That it’s not something to be silent about. …And I think it is wonderful.* [Individual interview]

Shared stories from participants inspired others to share personal issues in both formats. However, several noted that building the capacity and sense of security required for sharing could take time. Being open about personal issues in the ReConnect-cafés was said to be difficult, but since their need for personal sharing and support was met online, it was not considered a problem. One said:

*Everyone who’s there* [in the online forum] *understands that all of us struggle with a lot of stuff. And we understand that it can be difficult to talk about it. When we meet at a regular ReConnect*-*café, it’s not like we talk about that kind of thing. But when we have the forum to chat in, then we can come a little closer to the core.* [Individual interview]

Another reported that the experience of trust among the participants allowed more openness regardless of format:

*It [access to combined online and offline peer support] has led to me getting many good friends…It means that I can be open when I meet them * [at ReConnect-cafés]. *I feel like I have people I can trust. People I can talk with, also when I meet them other places. And in a totally different way than before.* [Individual interview]

.

Overall, participants appreciated having the opportunity to share sensitive and personal issues, both in the forum and in the ReConnect-cafés. They described hopes of maintaining the quality of self-disclosure and openness in their future relationships. One of the participants elaborated:

*We’ve agreed that after the project ends, outside of ReConnect, that we can still meet and share. Not just pleasantries, but we can also share when we aren’t doing so well. At the same time, we agree that if you don’t have a good day, it’s OK to say that today isn’t a good day to meet. We’ve talked a lot about that. Then we can also have days when we need to be able to share troubles, and that the others can say supportive things… You can fish a bit… “Are you OK today?” And then the other can say: “No, I didn’t sleep well last night”… and then we can talk a little about that.* [Individual interview]

### Enabling connectedness

The second main theme encompasses participants’ descriptions of how combining online and offline peer support groups facilitated connections with one another and the local community, but also posed some challenges.

#### New friendships

The friendships that evolved through online and offline peer support was described by participants in both communities as one of the most important benefits of participation in the study—“*it meant the world to me * […]* probably the best help I could get*”, one said. Several expanded their networks, and for one who had recently moved to the community, ReConnect enabled making her first friends there. Participants described that the friendships developed through ReConnect were unique due to common experiences of having mental health problems. They characterized these friendships with words such as recognition, understanding, fellowship and joy, and that they were qualitatively different from other relationships. One participant described her friendships through ReConnect with these words:

*I do have friends outside* [of ReConnect], *but it’s good that ReConnect is there. Because there I have people who understand me and what I struggle with. People who understand how it is if I relapse… It’s not certain that those (other) friends of mine understand me as well as those in ReConnect do, because they haven’t experienced the same things that I have. I feel that I’ve got very close friends now thanks to ReConnect. Both those I met there* [in the online forum], *but also in daily life.* [Individual interview]

Meeting each other face-to-face in ReConnect-cafés was described as important in facilitating a sense of kinship and empathy among the participants, as illustrated by the following quote:

*Everyone has been able to tell their story. And you’ve seen the face of the person sitting there talking. So, you get to know each other in that way as well. And then you think, “Wow, that person struggles with a lot of the same problems that I have”. So, you feel really…together. It makes me feel really warm in my heart.* [Individual interview]

The service user consultant in the study was described as essential for the participants’ positive experiences of combining online forum and the ReConnect-cafés. In a conversation about how the ReConnect-cafés were experienced as safe and useful, one participant noted that online facilitation by the service user consultant played a positive role:

*The forum is what is important for me. Lillian* [the service user consultant] *gives us exercises, things we can reflect over before* [meeting in] *the cafés. I don’t think* [the cafés] *would be the same without the forum.* [Individual interview]

#### Engagement in the local community

Participants described that the study contributed to increased engagement in the local community. In addition to study-initiated activities such as ReConnect-cafés, participants also initiated gatherings themselves, such as a spontaneously meeting for coffee, or more planned involvement together in activities organized in the local community. The online forum in ReConnect was used to exchange reminders and encourage participation in the various local activities. Some reported planning to volunteer to help refugees in the community, while one started organizing support for establishing a local service user organization so people with mental health problems can meet. The process of becoming more active together with people in the community was described by one of the participants as a process of “*getting out of hibernation*” and by another as “*things that contribute to happiness*”.

While the face-to-face ReConnect cafés in the local community were described as supportive and caring, both practical and emotional barriers to participation were also reported. One described feeling insecure about participating at gatherings with people she did not know, while another pointed to practical obstacles for attending such as dependency on others for transport. Another described discomfort even though she had been to ReConnect-cafés before and that she knew other people that would be attending at local activities. One reluctant participant reported giving into peers’ persistent solicitations about attending at a local arrangement:

*I’ve got such a nervous stomach that just going to the store, or filling the car with gas, I had to run to the WC probably 4*–*5 times before I got out the door.* […]. *And I told you that I wasn’t sure I’d get there because I dreaded it so much. Dreaded and dreaded and dreaded. But then you* [another participant] *sent the message yesterday, and then I thought, yeah, I’ll just say yes*. [Focus group]

The persistent peer referred to in the above quote acknowledged that she herself might have strengths that could be important in helping others, something she had not considered before. Similarly, participants also reported meeting before ReConnect-cafés, to help each other build courage to attend at ReConnect-cafés. The blended online and offline peer support was referred to as vital in lowering the threshold for engaging in meaningful daily activities as described above (e.g. volunteering for refugees, meeting for coffee), and even the process of getting back to work. The mutual support and “cheering for each other” was described as invaluable. As one participant who succeeded in returning to part-time work explained:

*It’s sort of been alpha and omega for me. Having that support at my back* [online and offline peer support], *that’s what made it possible for me to get back into my life as well as I have now. It’s been nothing but positive.* [Individual interview]

The face-to-face relationships that evolved during the course of the study were valued to the extent that many started working on ideas for projects that could help them stay in touch. One idea was to get together to run a property that someone had donated to the municipality for mental health purposes, as illustrated by one participant:

*We don’t want to lose each other. So we’ve been discussing what we can do. We’ve got lots of ideas. Someone* [in community] *donated a piece of land with a pond for a park; it just has to be used for something to do with mental health. So those who struggle with their mental health can meet, be there, bring their families and that sort of thing.* [Individual interview]

This quote is also illustrative of many other similar expressions of reciprocal support, acknowledgement of ones strengths to others, and a collectiveness that together signaled a sense of empowerment.

## Discussion

### Principal findings

To our knowledge this is the first study to systematically explore the benefits and challenges of combining online and offline peer support groups as an adjunct to ongoing mental health care for people with long-term mental health problems. Prevalent throughout the findings were known features of online peer support such as recognition, acknowledgement and self-disclosure [40]. It appeared important that both formats were peer moderated and offered outside the context of mental health services and its health providers [33, 66]. In addition, the analysis identified a number of benefits and challenges of combined online and offline peer support. The participants’ descriptions of their experiences touched on two main themes: (1) balancing anonymity and openness, and (2) enabling connectedness. Three of the four subthemes mainly describe benefits, while challenges were less clearly stated and cut across the subthemes. Identified challenges were linked to transitioning between anonymity and being known in-person, how to protect confidentiality, and issues related to participation in offline peer support groups in the local community.

The first theme, balancing anonymity and openness, indicates that the open and non-judgmental atmosphere found in anonymous peer support groups [38, 39] may be enhanced by combining online and offline peer support groups. This is illustrated by how self-disclosure and openness in one format could migrate and be reinforced toward greater openness about personal issues in the other format. The mutual self-disclosure in an anonymous and secure online environment appeared to reduce a sense of stigma and fostered the trust necessary to muster courage to self-disclose when also meeting face-to-face. As others have found, some find it easier to express ones’ ‘true-self’ online [41], and being accepted for who one is online can reduce fear of acceptance offline. Both group formats appeared to facilitate the sharing of personal stories that participants characterized as instilling hope and inspiration, reflecting support for an essential dimension in personal recovery [10]. The combination of formats opened for more opportunities for discovering, for example, that helping another participant get to the ReConnect-café revealed one’s own strengths which could in itself create hope and inspiration, also referred to as helper therapy [18]. In sum, these experiences can be said to promote an identity as “normal”, rather than as a “service user” or “patient”, which many find pacifying and/or stigmatizing [10]. In addition, the service user consultant served as a role model for participants when moderating the peer support groups, while she identified and praised respective peers’ personal strengths. Discovering that one is not alone, or not so different from others, and that others value one’s viewpoints is also inherent to peer support regardless of format.

The second theme, enabling connectedness, comprises of participants’ descriptions of new friendships and engagement in the local community, reflecting a vital dimension in recovery [10]. The analysis revealed a sense of belonging or connectedness among participants that could have been achieved through one of the formats alone, but that appeared strengthened or amplified by combining them. The very nature of peer support groups, whether offered online or offline, fostered connectedness through the common frame of reference that members now shared. In line with recovery-oriented approaches [67], this common frame of reference was not a specific diagnosis, or formal status as patient, but rather common life experiences related to striving towards fulfilling lives regardless of what symptoms they may have. This second theme, enabling connectedness, suggests that combining online and offline peer support groups opened new paths towards friendships and engagement in the local community. While knowledge about how online peer support relationships migrate to in-person meetings is scarce [46], the combination of formats appeared to facilitate friendships and in-person engagement in the local community that were otherwise unlikely. Our findings suggest that the risk of online formats undermining face-to-face relationships, particularly for those with social anxiety [36], might be counteracted by explicitly using the online format to facilitate in-person encounters. Connectedness through the combined formats was reportedly instrumental in regaining employment for one participant, suggesting at least a potential for the types of tangible improvements that others have found elusive [46] are possible. One study suggest that online peer support offers help for specific questions such as housing and employment [33]. The participants’ reciprocal focus upon personal strengths and what they could do collectively, as well as their engagement in community activities or the job market appeared to be empowering and meaningful, both of which are predictors of recovery-oriented outcomes [68].

The findings in this study also reflect challenges related to combining the peer support formats. Participants’ commitments to preserving confidentiality, while at the same time weighing exposure of one’s own identity online, was reported as challenging and, at least initially, a deterrent to self-disclosure. Online anonymity was also described as being less genuine and hence limiting a sense of connection and community. A recent study also reflects on similar dilemmas related to solely online support groups [33]. In this study anonymity is described as a “double-edged sword” (p. 7) in the sense that it allowed the users to let more out, but at the same time they had to be careful about who knows who you are in the offline world. For those who value online formats because of their social anxiety, or concern with stigma, such considerations may well deter participation in the offline group [36]. Participants who resolved these types of dilemmas did so differently, but in ways that appeared to be in line with their own values and comfort zones. As others have found [69], participants appreciated having options (e.g. the choice of participating online, offline or in both group formats) that can be explored and tailored to their personal preferences.

### Limitations

A major limitation to this study was our failure to recruit male participants, despite considerable efforts to do so. In the mixed methods study only two of 29 service users were men, none of whom volunteered for this study. Possible explanations include the fact that all of the research team members were women and that apparent gender differences in e.g. online social activity [70] may have been at play. This study analyzed participant experiences with peer support groups that were part of a larger, complex intervention that included multiple online resources as an adjunct to ongoing care. We do not know if these experiences would have been different if the two group formats had been offered without the broader intervention components. We did not explicitly explore how the different settings might have influenced the findings, which might have added value to the study. The methods used in this study do not allow for immediate generalization of the findings, but the insights may have relevance to other contexts. The authors were involved in designing ReConnect as well as in generating data about its use. Efforts to reduce potential biases included inviting participants to give critical feedback both about the portal and our tentative data analyses, and by collecting the data over time. The inclusion criteria for the individual interviews (having logged on ReConnect > 15 times) favors those who actively used ReConnect, thus excluding those who may have neglected to use ReConnect due to negative attitudes or experiences.

### Implications for practice and future research

The findings in this current study indicate that combined formats for peer support groups enabled options that can represent a valuable resource in recovery-oriented services. In efforts to leverage the respective strengths of peer support groups in combined online and offline formats, some issues are worth attention.

The service user consultant played a critical role in this study not only as a peer in line with international recommendations [71] and research [37], but also as moderator and facilitator for both formats. Although we did not study this role explicitly, some observations are worth considering for future research and practice. Having a well-versed basis in recovery-oriented principles helped guide the moderator in responding to participants in positive and ethically sound ways. The relational continuity of having the same moderator for both formats, appeared to foster a sense of familiarity and security among participants, as well as positive synergies between the formats, in ways that may have been less likely had the formats had separate moderators. This issue is worth more attention in future studies. Guidelines that help participants anticipate challenges, e.g. in transitioning between levels of anonymity and in safeguarding confidentiality, need to be developed to support both moderators and participants. Combining offline and online peer support groups in adjunction to ongoing mental health care moderated by a service user consultant versed in recovery might be particularly well suited for engaging service users in the implementation of recovery-oriented care [51].

Although the forum functioned well in this study despite a small number of participants, a greater number of participants would be preferable in future practice and research. This may ensure a minimum level of activity necessary for maintaining interest in revisiting on a regular basis, as well as help ensure a breadth of experiences and perspectives. Also, a higher number of participants may protect the anonymity and confidentiality of individuals from the same community. Future research needs to address ways of facilitating the translation of social relationships in online and offline peer support formats into health-promoting relationships within local communities. Research into gender differences in recruitment and participation in peer support groups for both formats is also needed. Reasons for non-use should also be addressed in future research.

## Conclusion

This study suggests that online and offline peer support groups as an adjunct to ongoing mental health care for people with long-term mental health problems are complementary and that combining the two formats can facilitate social relationships, promote friendship and community connectedness. These benefits appeared to stem from the service users’ opportunity to choose between, or combine the two formats, according to their individual needs, values and comfort zones. The challenges identified were linked to transitions from anonymity to becoming identified, protection of confidentiality, and participation in offline peer support groups in the local community. Moderation of peer support by a trained service user consultant is suggested essential in both formats. Combining online formats that offer users round-the-clock access regardless of location, anonymity and a non-judgmental atmosphere, while at the same time fostering local, in-person community ties, appears to be a promising concept for facilitating recovery-oriented care and is worthy of continued research.

## Supplementary information

**Additional file 1.** Interview guide for focus groups.

**Additional file 2.** Interview guide for individual interviews.

## Data Availability

The datasets generated and analyzed during the current study are not publicly available due to considerations of confidentiality. Anonymized data (Norwegian only) are available from the corresponding author on reasonable request.
