# Supplementary material for: Combining online and offline peer support groups in community mental health care settings: a qualitative study of service users’ experiences
Source: Int J Ment Health Syst. 2020 May 29;14:39. doi: 10.1186/s13033-020-00370-x (PMC7260836; doi:10.1186/s13033-020-00370-x)
Supplement: Supplementary file 1 — Additional file 1. Interview guide for focus groups. [file 13033_2020_370_MOESM1_ESM.docx]

1. Let us please start with a short presentation of ourselves – first name, short about how you have used ReConnect and for how long.
2. Could each of you please say a word or two about your reasons for, and expectations towards, participation in the study?
3. Please share examples of how you have used ReConnect – both on your own and when you have used it together with your health provider.
4. What works well, and what would you like to be different? Examples are helpful.
5. Please share any thoughts you may have about how use of ReConnect could be more beneficial for yourself, and for your collaboration with your health provider, during the rest of the study period.
6. Do you have any questions or comments before we try to summarize what we have talked about?
