# Supplementary material for: Combining online and offline peer support groups in community mental health care settings: a qualitative study of service users’ experiences
Source: Int J Ment Health Syst. 2020 May 29;14:39. doi: 10.1186/s13033-020-00370-x (PMC7260836; doi:10.1186/s13033-020-00370-x)
Supplement: Supplementary file 2 — Additional file 2. Interview guide for individual interviews. [file 13033_2020_370_MOESM2_ESM.docx]

1. Could you please start with a short presentation of yourself – first name and how long you have used ReConnect?
2. If you participated in a focus group earlier, has anything changed with regards to how you use ReConnect since then? How?
3. Can you tell more about how you and your health provider collaborate by using ReConnect? What works well and not so well? Examples are helpful.
4. Please describe more about how you use ReConnect on your own. What works well and not so well? Examples are helpful.
5. For service users only: Please describe how you have experienced participating in the discussion forum and/or the ReConnect-café.
6. Has ReConnect played a role for you personally, and/or in how you work with your health provider (both positive and negative)? Please describe.
7. If ReConnect were to become an ordinary service after the study period, what would be important in order for you to benefit from it?
8. Before we end, do you have any questions or comments on what we have talked about?
